# Research in Academia: Creating and Maintaining High Performance Research Teams

**DOI:** 10.1155/2019/8423460

**Published:** 2019-02-03

**Authors:** Maria Olenick, Monica Flowers, Tatayana Maltseva, Ana Diez-Sampedro

**Affiliations:** Nicole Wertheim College of Nursing and Health Sciences, Florida International University, Miami, FL, USA

## Abstract

As universities strive to raise their academic rank through the quality and quantity of scholarship in order to maintain their competitive edge and funding sources, faculty face pressure to increase number of publications and externally funded research (or project proposals). There are many challenges that make it difficult for faculty to meet a university's research demand, such as increased work load in academia, teaching large-size classes of students, and other strict university deadlines related to book ordering, scheduling classes, posting grades, etc. Faculty work group conflicts, faculty incivility, and dwindling grant/research funding add to faculty stress. In order to promote scholarship in academia, administrative support, collaborative work environments, mentoring, and appropriate appraisal systems are needed to enable faculty to be more productive and satisfied.

## 1. Introduction: Research and Scholarship in Academia

In a rapidly changing world, it becomes imperative for faculty and students to remain innovative and competitive. Research builds knowledge through dissemination of valuable information, facilitates learning, increases awareness of various issues, proves/disproves lies versus truths, opens opportunities, and exercises the mind [1]. The United States' (US) higher education system commemorates research-intensive institutions and furthermore ensures that they “receive a share of funding from state and local appropriations” [2, p.1].

As universities strive to raise their academic rank through the quality and quantity of their research and scholarship in order to maintain their competitiveness and funding sources, faculty face pressure to increase number of publications and external funding through grant proposals. As a result, academic appraisal systems shift towards rewarding research more than teaching in order to increase university rankings. This leaves many faculty “to neglect teaching to focus on producing research”, spending time on research allowing “for less time to concentrate on teaching”, and publishing many low-quality, low-value papers in order to meet the demand [3, p. 2]. Unfortunately, faculty must oblige to the appraisal systems' criteria as it is critical for keeping their jobs and for being promoted to a higher rank. Furthermore, appraisal systems determine whether a tenure-earning professor earns tenure at their review.

## 2. Work Group Functioning and the Nursing Faculty Shortage

Shortages of nursing faculty already limit student enrollment capacity across the country [4]. Shortages of nursing faculty, particularly PhD prepared research faculty, limit potential for funded research at universities [5], which can affect university research designation. As faculty work individually in their attempt to meet desired publications, grant proposals, and funding expectations in light of the national nursing faculty shortage, they begin to realize that functioning alone is extremely difficult. Effectively creating and maintaining productive work groups where everyone contributes can open far more possibilities and opportunities for productivity. Combining faculty who are strong writers, faculty who are strong in editing, and faculty who are strong in research methods and data analysis establishes a research and scholarship “machine” where faculty are better able to submit higher quality manuscripts and funded proposals at a much more desirable pace. Combinations of varied faculty in work groups also ensure internal preliminary peer review since multiple sets of eyes on a document are better than one person's eyes alone. Optimizing productivity from faculty through effective work group encouragement is necessary in this time of faculty shortage.

Many initiatives are aimed at addressing the nursing faculty shortage. American Association of Colleges of Nursing (AACN) [4] lists several on their website including, but not limited to, the following: the Jonas Nurse Leaders Scholar Program, which provides funding and support to doctoral nursing students with the hope that they will become nurse educators and researchers; the Johnson & Johnson Campaign for Nursing's Future which is aimed at diversifying nursing faculty; the Nurse Faculty Loan Program by Health Resources & Services Administration (HRSA) which is a loan forgiveness program for qualified nurses in an advanced education nursing degree program(s) to serve as nurse faculty; and the Graduate Assistance in Areas of National Need (GAANN) which is a funding stream for nurses to achieve a PhD. All of these initiatives hope to increase and improve the educational level of nurses prepared for the task of scholarship related activities such as grant writing and publishing as nurse faculty at academic institutions.

Effective work groups, including interprofessional healthcare groups, can create a “divide and conquer” creative capacity that working alone cannot capture. Being part of a group that is working towards common goals, even when working alone, may also be motivating to individuals [6].

## 3. Conflict within Work Groups

The intention of work groups is to collaborate and to carry the collegial spirit forward in creating and disseminating new knowledge. Unfortunately, some projects do not make it to publication or grant submission due to disagreements within teams, loss of motivation, or faculty moving on to new projects without finishing and publishing what was started. Well-meaning ventures can lead to faculty conflict for various reasons. Faculty working on collaborative projects might be aware that some conflict is expected, but with this challenge comes an opportunity to better understand human psychology and resilience. Tekleab and Quigley [7] identified that team members who are conscientious, emotionally stable, and respectful of other team members' values are better able to resolve conflict and prevent negative reactions from spreading due to conflict. Optimizing team conflict dynamics relies on a productive team design and an organizational environment that values and supports high performance teamwork [8].

Another factor that prevents successful grant submission or publication is loss of faculty motivation while working on a project. Faculty might experience limited understanding, resources, and expertise and, therefore, will cease working on a project before completion. Establishing collaborative partnerships with other disciplines and working together facilitates the development of new forms of creativity and innovation and generates faculty's self-confidence and improves the team's outcomes. Collaboration is beneficial as it permits faculty to accomplish more than they can individually, to reach out to people and communities, and to grow professionally [9]. A motivational leader in the group can also facilitate the productive completion of research and publication.

Lastly, a final factor preventing successful grant submission or publication is the inability of faculty to complete a project on time before moving on to the next project or work assignment. The literature supports that faculty often feel overwhelmed with the increased work load in academia, teaching large-sized classes of students, and meeting other projects and strict university deadlines [10–12]. Faculty are under the pressure to move students through the curriculum, graduate students on time, and prepare them to pass licensure and certification exams on first attempt. Consequently, this pressure could be a reason why faculty stop working on manuscripts or research. The demands on faculty to conduct research and publications are very time consuming, thus creating competing demands for limited amounts of time and effort on the part of the faculty such as ordering books and posting grades on time. Faculty burnout in academia may be prevented by providing administrative support to increase faculty productivity and quality of their scholarship [11]. Faculty's engagement in scholarship activities provides insight into the support provided by the university and its administration, and their understanding of the role of the faculty in teaching, research, and service.

## 4. Faculty Incivility

Academic environments can be extremely competitive and stressful secondary to pressures to consistently produce scientific and scholarly work despite nursing faculty shortages. Also, inflation-adjusted research funding by the National Institutes of Health (NIH) has dwindled over the years, making it increasingly difficult for nursing faculty, thus possibly causing tempers to flare for fear of losing positions or status [13]. Control, power, and jealousy may all contribute to incivility among nursing faculty [14].

Most colleges of nursing would benefit from senior faculty helping junior faculty to grow, however, there is often a lack of mentorship and a culture of withholding information or direction that could make another faculty successful or outshine a more experienced or senior faculty [15]. It is in these instances that incivility such as alterations in communication or mentorship may be first recognized.

## 5. Recommendations: Creating and Maintaining High Performance Research Teams

In order to produce quality research while maintaining excellence in teaching, a balance must be found. Support from administration is of upmost importance via (a) course release for research, (b) distinct appraisal systems for clinical/teaching versus tenure/research faculty, (c) teaching assistants (TA) to offset the work and concurrently TAs receive research mentoring, (d) providing funds for pilot studies, and/or (e) decreasing the quantity of required publications to promote higher quality work. A strong mentoring program for new/junior faculty led by senior professors, writing workshops headed by research-focused faculty, research support infrastructure, content-focused think tanks, and intradepartmental collaborative work to disburse workload are also needed. Those that are focused on and excel at teaching can focus their publications and research on their teaching activities.

In addition to their role in faculty improvement, appraisal systems have the potential to be punitive. If appraisal criteria are not met, then the individual is not promoted or retained. As we learned from Olds and Milner [16], positive reinforcement will make it more likely that a behavior will occur again in the future. Thus, it becomes imperative for administrators and faculty to work together to find a balance between research and teaching, for example, faculty that meet appraisal criteria: pick their course assignment, have a reduced workload, receive an award luncheon or the college makes an announcement regarding their work. These reinforcements are more likely to contribute to a culture of productivity and employee satisfaction. On the other hand, those that do not meet criteria are paired with a mentor, given constructive criticism, and developing an action plan for success.

## 6. Conclusion

As nursing faculty are increasingly asked to perform teaching, research, and service, the demands to perform at a high level in all areas are subject to competing demands for time and attention. Coupled with a challenging and very limited funding environment, the need to implement team-based research groups becomes more evident. By developing working research teams of faculty who can each use their strengths to contribute to a common goal, faculty motivation and achievement in research can be improved. A well-qualified and motivational group leader is generally needed to facilitate the performance of teams, and to ensure that team members are recognized for their contributions to the goal.

In addition to the implementation of team-based research, university administrations should have at their disposal the tools to contribute to faculty success, for example, implementing distinct assessment criteria for different faculty tracts and supporting their goals by developing formal mentoring programs, among other policy changes like faculty assessment optimizations. Faculty assessment has the potential for acting punitively, but through smart policy decisions and development of support mechanisms for faculty who do not meet goals, administrators have the potential to develop an army of qualified researchers and educators who can support the goals of their universities.

## References

[1] Zarah L. (2018). *7 reasons why research is important*.

[2] American Academy of Arts & Sciences (2015). *Public research universities: Why they matter*.

[3] Lai M., Du P., Li L. (2014). Struggling to handle teaching and research: A study on academic work at select universities in the Chinese Mainland. *Teaching in Higher Education*.

[4] American Association of Colleges of Nursing (AACN) (2017). *Nursing faculty shortage*.

[5] McDermid F., Peters K., Jackson D., Daly J. (2012). Factors contributing to the shortage of nurse faculty: A review of the literature. *Nurse Education Today*.

[6] Carr P. B., Walton G. M. (2014). Cues of working together fuel intrinsic motivation. *Journal of Experimental Social Psychology*.

[7] Tekleab A. G., Quigley N. R. (2014). Team deep-level diversity, relationship conflict, and team members' affective reactions: A cross-level investigation. *Journal of Business Research*.

[8] O’Neil T. A., McLarnon M. J. (2018). Optimizing team conflict dynamics for high performance teamwork. *Human Resource Management Review*.

[9] Green B. N., Johnson C. (2015). Interprofessional collaboration in research, education, and clinical practice: Working together for a better future. *Journal of Chiropractic Education*.

[10] Bittner N. P., Bechtel C. F. (2017). Identifying and describing nurse faculty workload issues: A looming faculty shortage. *Nursing Education Perspectives*.

[11] Candela L., Gutierrez A., Keating S. (2015). What predicts nurse faculty members' intent to stay in the academic organization? A structural equation model of national survey of nursing faculty. *Nurse Education Today*.

[12] Lee P., Miller M. T., Kippenbrock T. A., Rosen C., Emory J. (2017). College nursing faculty job satisfaction and retention: A national perspective. *Journal of Professional Nursing*.

[13] Federation of American Societies for Experimental Biology (FASEB) (2018). *NIH Research Funding Trends*.

[14] Clark C. M., Olender L., Kenski D., Cardoni C. (2013). Exploring and addressing faculty-to-faculty incivility: A national perspective and literature review. *Journal of Nursing Education*.

[15] Dunham-Taylor J., Lynn C. W., Moore P., McDaniel S., Walker J. K. (2008). What goes around comes around: Improving faculty retention through more effective mentoring. *Journal of Professional Nursing*.

[16] Olds J., Milner P. (1954). Positive reinforcement produced by electrical stimulation of the septal area and other regions of rat brain. *Journal of Comparative and Physiological Psychology*.

